# Design and evaluation of an immunology and pathology course that is tailored to today’s dentistry students

**DOI:** 10.3389/froh.2024.1386904

**Published:** 2024-05-09

**Authors:** Teun J. de Vries, Ton Schoenmaker, Laura A. N. Peferoen, Bastiaan P. Krom, Elisabeth Bloemena

**Affiliations:** ^1^ Department of Periodontology, Academic Centre for Dentistry Amsterdam (ACTA), University of Amsterdam and Vrije Universiteit, Amsterdam, Netherlands; ^2^ Pathology, Amsterdam UMC Location Vrije Universiteit Amsterdam, Amsterdam, Netherlands; ^3^ Oral and Maxillofacial Surgery and Oral Pathology, Amsterdam UMC Location Vrije Universiteit Amsterdam, Amsterdam, Netherlands; ^4^ Department of Preventive Dentistry, Academic Centre for Dentistry Amsterdam (ACTA), University of Amsterdam and Vrije Universiteit, Amsterdam, Netherlands; ^5^ Academic Centre for Dentistry Amsterdam (ACTA), University of Amsterdam and Vrije Universiteit, Amsterdam, Netherlands

**Keywords:** immunology, pathology, dentistry, education, curricular reform, teaching methods, ChatGPT

## Abstract

Curricular reform provides new opportunities to renovate important pillars of the dentistry curriculum, such as immunology and pathology, with novel approaches that appeal to new generations of students. When redesigning a course that integrates both immunology and pathology at the level that provides dentistry students with sustainable knowledge that is useful for their entire career, several challenges must be met. The objective of the present study was to describe the considerations involved in the design phase of such a new course. First, the course should be compatible with the new view on the incorporation of more active learning and teaching methods. Practically, this means that the course design should contain fewer lectures and more seminars and tutorials, where the students have fewer contact hours and actively engage in using recently acquired knowledge within a contextual background. A mandatory session of team-based learning provides opportunities to apply knowledge in combination with academic reasoning skills, teamwork, and communication. Second, for a 4-week course, choices must be made: students will not become immunologists nor pathologists in such a short period. A governing principle for this course's design is that it should be based on understanding the basic principles of immunology and pathology. The ultimate goal for the students is to make the course immuno-*logical* and patho-*logical*, challenging them to reach a next level but clearly without oversimplification. Part of the course design should allow room for students to further study an immunological topic of their own choice, thereby contributing to their immunological curiosity and to their academic development. Third, to make it tailored to a new generation of dentists, examples from the field of dentistry are actively integrated in all aspects of the course. Finally, the era of ChatGPT provides novel opportunities to use generative artificial intelligence (AI) tools in the learning process, but it demands critical thinking of how to use it in a newly designed course. A mid-course evaluation revealed that students acknowledged that immunology and pathology were presented as an integrated course. The final course evaluation endorsed the use of these various educational methods. These methods proved to be appropriate and logical choices for reaching the learning goals of the course.

## 1 Introduction: background and rationale for the educational activity

A new curriculum has been implemented for the bachelor program of the Academic Centre of Dentistry Amsterdam (ACTA), Netherlands. Key innovations contain a clear alignment (theory–preclinical skills–clinic), earlier acquisition of basic skills, more integration of dentistry aspects in basic science courses, and a general redefinition of basic science courses. Within the 3-year Bachelor's program, the emphasis is much on basic sciences and prepares for clinical skills that are needed in the 3-year Master's program. One decision connected to the reform of the Bachelor's program has been to split a large course of Infection and Inflammation of 8 European credits (EC; where 1 EC = 28 h of study) into two smaller courses. Infection and Inflammation contained aspects of microbiology, hygiene and infection prevention, gingivitis, immunology, and pathology where teachers from at least four departments were involved in teaching. The Infection and Inflammation course contained too many topics and many students failed this course at the first attempt. This larger course was split into two courses: Microbiology for Dentistry (a bachelor-1 course) and Immunology and Pathology (a bachelor-2 course), each course with 4 EC. Such a split aimed at more coherence within each course. The positioning of the course in the second year of the Bachelor’s program seems the right place, since it builds on fundamental courses: Cells and Tissues; Microbiology for Dentistry; Organs and Systems; and Cariology and Oral Biology. As such, it provides the basis for Periodontology I and Periodontology II as well as for The Complex Dentistry Patient, which are taught later in the curriculum.

Parallel to redefining courses was the implementation of active teaching and learning educational forms. Active learning methods have shown to be successful in meta-analyses: the learning process was better and the percentage of passed exams increased accordingly (1, 2). A meta-analysis that specifically analyzed active learning strategies among dentistry undergraduates across 93 studies concluded that active learning improved satisfaction and knowledge acquisition, and was rated as superior to traditional teaching and learning methods (3). Lecturing large audiences is effective for presenting information, but such an educational choice is less effective for stimulating higher-order thinking (4).

## 2 Pedagogical framework(s), pedagogical principles, and competencies/standards underlying the educational activity

To increase the frequency of active learning activities, the educational directors of ACTA made it mandatory to reduce traditional lecturing to the whole cohort of 144 students in large lecture halls to a minimum. Other implemented learning methods include tutorials for 2 h per half of the cohort (groups of 72 students) and seminars for 2 h per week for groups of 24 students. Both lectures and tutorials were advised to contain interactive aspects, such as quizzes, and to apply a questioning approach to further involve students. For the design of tutorials, teachers were asked to build in specific interactions with students, for instance by addressing the class with questions during the tutorial. For the design of seminars, students are in the lead. Assignments are made in class within smaller groups. Teachers are there to facilitate the process and to present extra information, supplementing the assignments in class. Finally, each course must have at least one team-based learning (TBL) session. Team-based learning is an active learning method, widely used in the medical curricula, where it was shown to improve performance (5, 6). With TBL, students spend 1 day preparing for individual readiness assurance tests (iRAT), where approximately 20 multiple-choice questions are answered, followed by answering the same questions as a team (tRAT). Subsequently, teachers prepare a mini lecture to explain common mistakes in the iRAT/tRAT. After the RAT stage, teams of five to seven students take part in application sessions where approximately three application questions are answered. Typically, students choose one of four outcomes and are able to defend this choice. Possible answers are phrased in such a way that they elicit discussion among students in which they actively apply knowledge obtained during the previous teaching activities. With TBL, higher levels of learning are achieved, fostering the development of clinical reasoning skills early in the career of a future dentist.

For the course design, the following principles were leading.

### 2.1 Immunology and pathology topics should be presented as complementary and integrated entities

**Figure 1:**
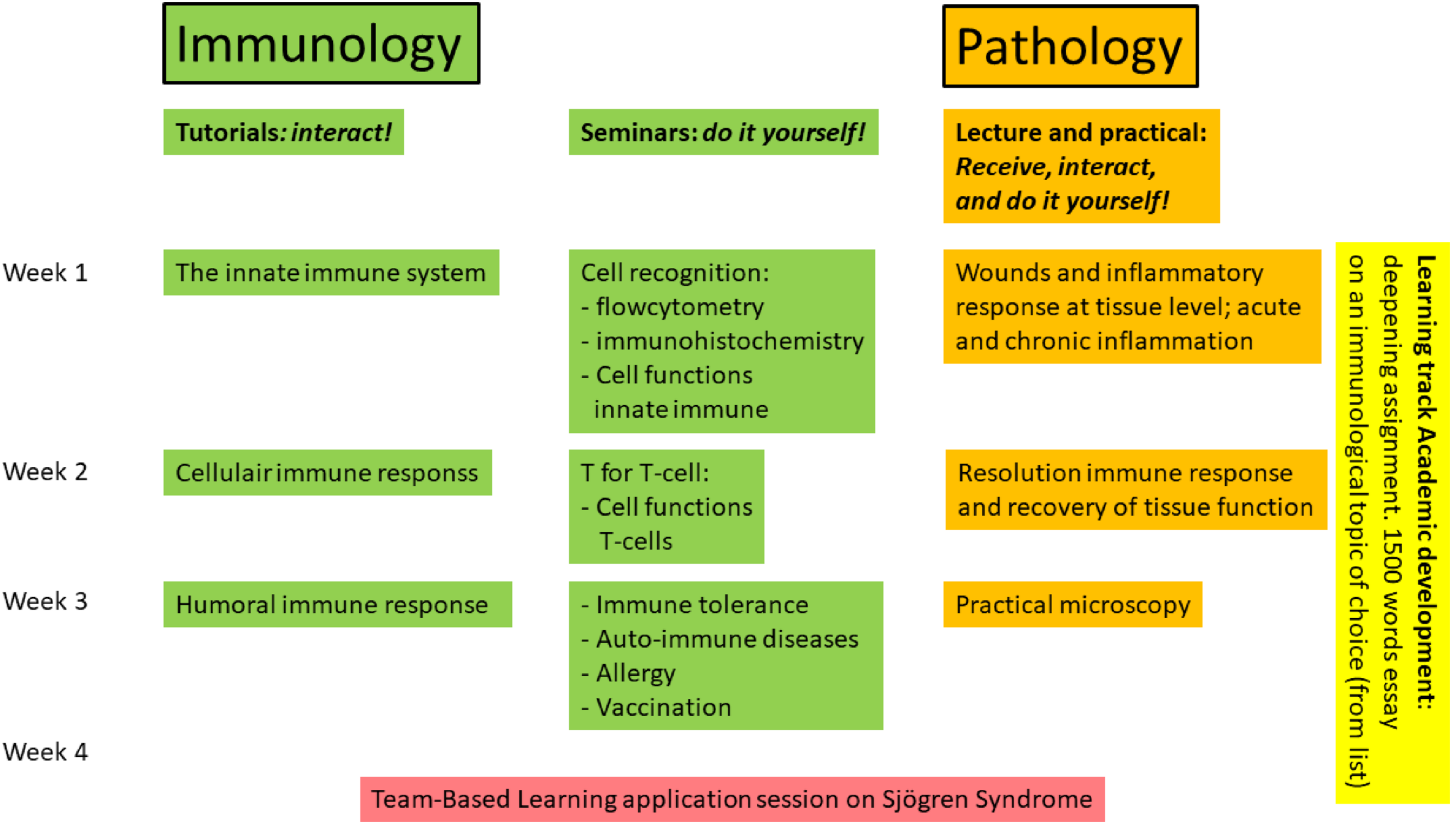
Course design per week with the various themes per week. Green indicates Immunology, orange Pathology, and team-based learning in red is both Immunology and Pathology. Immunology was taught in tutorials and seminars and Pathology in lectures and using an interactive practical. Each week had a theme, and per week, tutorials, seminars, and lectures were followed in a logical sequence. The Learning track Academic development in yellow is overarching the 4-week period. Team-based learning functioned as a further bridge, evoking academic reasoning on various aspects of Sjögren’s syndrome.

To avoid the pitfalls of the previous, longer course, we developed the shorter course to possess a more logical sequence and greater integration between subjects. In other words, we aimed for coherent crosstalk between immunology and pathology. Ideally, each in-class contact should pick up where the other had left off. The immunology seminars should follow logically on the tutorials, and the pathology lectures at the end of the week should subsequently follow logically on what was learned at the immunology seminars and tutorials (see Figure 1). While the emphasis of immunology was much more on the cellular processes, the pathology sections focused on the governing principles within tissues, with emphasis on the molecular and tissue response in acute and chronic inflammation and the recovery phase. Next, a clear division of teaching activities was incorporated: tutorials and seminars for immunology (TdV and TS); and lectures and practical for pathology (EB). A team-based learning application session was organized around this theme as the final educational activity, requiring the application of previously acquired knowledge, and thus promoting a higher level of learning. A theme on an autoimmune disease, Sjögren's syndrome, which is relevant for dentists-to-be, provided a synthesis of both immunology and pathology. It contained questions on designs of pathological immunohistochemical stainings to learn more about the immune cell infiltrates that are typical for Sjögren’s syndrome (7), a question on Sjögren and parameters associated with periodontitis (8), and a question on ranking the most effective treatment (anti-tumor necrosis factor (TNF); anti-B-cell, anti-autoimmune-B-cell) based on a table in the study by Kroese et al. (7).

### 2.2 Define the boundaries for the course of immunology and pathology, but allow for further exploration

The scientific fields of both Immunology and Pathology have tremendously expanded in recent decades. Therefore, it was important to set the boundaries of what should be learned. A central question was: to what extent should details be memorized? Often, textbooks contain too many details. Therefore, our starting point was that mechanistic insight matters. Acute and chronic inflammation is the first line of defense against loss of tissue homeostasis and part of the innate immune system. For example, if students are able to link innate immunity with cellular immunity and that T-helper cells help enforce certain innate immunity responses, students make the connection. Students should also actively categorize cellular entities, such as the two major possibilities to combat pathogens in innate immunity, being either phagocytosis or, if the parasite is too large, making holes. Finally, they should emphasize similarities, for instance between cytotoxic CD8 cells and Natural Killer (NK) cells. Choices for a relatively short course meant for immunology to emphasize the triad innate immunity, the cellular immunology of the acquired immune system and humoral immunology. After seeding this core knowledge, complementary topics for the last week were tolerance, autoimmunity, allergy, and vaccination. The duration of the course did not allow for explaining tumor immunology and transplantation immunology. Such topics and their relevance for the dentistry field are addressed in courses later in the curriculum.

For a student who should know core concepts and mechanisms, the course, especially the immunology part, invites further reading and specialization. Part of the curricular reform at ACTA is a Learning Track Academic Development (BK) which is spread over the whole 3 years of the Bachelor phase and continued in the Master phase. During the 3 years, students prepare for one of their end-products, which is a Bachelor's thesis written in duos. In the first year, they acquire the basis of scientific writing, ethics and philosophy of sciences, methodology, and statistics. The aim of the second year was to practice their acquired writing skills, which were integrated in this course. In parallel to the 4-week course, students had to choose an immunology-driven topic from a list (Table 1) and were asked to write an essay of 1,500 words. As can be observed in Table 1, the topics of choice were formulated in a rather general way, leaving space to approach these topics in a more specific way. They were explicitly given the option to use generative artificial intelligence (AI) in the process and given clear guidelines on transparency on how and where they used it. Before their assignment they all followed an E-module on the use and limitations of AI. The assignment was performed in duos to stimulate discussion and collaboration and was graded on technical writing skills rather than on immunological content. However, the in-depth literature searching, reading, and synthesis of logic and thoughts on immunological aspects supported deeper learning. The academic skills practiced here and elsewhere are instrumental for the efficient and proper preparation of a Bachelor thesis during their third year.

**Table 1:** Topics and research questions for essays.

Topic	Research question
1. Probiotics and the immune system	What are possible mechanisms on how probiotics can improve the immune system?
2. Prebiotics and the immune system	What are possible mechanisms on how prebiotics can improve your immune system?
3. Immune aging	How does age influence your immune system?
4. Sex and immune system-1	Based on the number of X chromosomes (males 1, females 2): how can you explain sex-skewness of immunological phenomena?
5. Sex and immune system-2	What is the role of sex hormones on the immune system?
6. Immune fitness	Immune fitness is a relatively new term in dentistry literature. What is it and how can one assess it?
7. Vaccines	Both SARS-CoV-2 and the HIV virus are frequently mutating viruses. Explain why vaccines against SARS-CoV-2 were immediately successful, whereas there is still not a vaccine against HIV
8. Stress and the immune system	Chronic stress induces the production of more stress hormones. What evidence is available that high levels of stress hormone lead to a worse function of the immune system?
9. Anti-inflammatory: good for periodontitis?	Anti-TNF medication is common for rheumatoid arthritis patients. This could be beneficial for the periodontal status of these patients. What is the evidence?
10. Nickel allergy	What components of the immune system are involved in nickel allergy?
11. The inflammatory osteoclast	Chronic inflammation, such as periodontitis, is associated with high levels of IL-1β and TNF-α. What is the role of these cytokines in the activation of osteoclasts?
12. Th17 and candidiasis	What are the immunological explanations for increased candidiasis in patients with IL-17 receptor mutations?
13. ACE-2, SARS-CoV-2, and oral health	One of the most cited articles in dentistry is the high expression of ACE-2 on oral epithelium. SARS-CoV-2 binds to cells with this receptor. What is known about the degree of infection of oral epithelium and the response of the immune system to this?
14. SARS-CoV-2 and salivary glands	What makes the salivary glands a good niche for this virus?

### 2.3 Themes per week, be clear about the readings

We decided for the immunology part to select readings of a Dutch textbook that is used in many medical faculties and at the Dentistry Faculty in Groningen (9). When applying the guidelines on what is feasible per hour of reading (6 pages of an information dense textbook), one should make a list of selected readings of the book. Reading and before-class preparation also contained readings of selected case reports related to dentistry (see Section 2.5). Since immunology is a topic that inevitably contains a lot of cartoons on working mechanisms, a list of important figures was provided as a learning aid. An action point that logically followed from a mid-course evaluation was to provide a list with core concepts that also included a list of cytokines that should be known. For a 4-EC course, it is far too much to learn all transcription factors by heart for all the immune cells. It is, however, important to know the function of these cells and where and how their differentiation takes place, explaining the logical sequence of the differentiation.

There are many explanatory and educationally well-designed clips on the internet (e.g., YouTube) that could be of aid, but when providing detailed boundaries of the course, many of those clips are confusing. Some of them are well-meant but oversimplified, and others contain far too much detail. For the course, we therefore limitedly suggested clips from the Internet.

For the pathology part, the selection of inflammation and tissue repair are relevant and, as the innate response, interact with the immunology part. Suggested readings were defined from a widely used international pathology book, which is updated on a yearly basis (10). Next to that, references were made during lectures to well-designed clips on YouTube.

### 2.4 Incorporate various educational methods to support the learning goals and objectives

When designing a course, it is important to use the constructive alignment as described by Biggs and Tang (4). This requires careful consideration of what to use for what learning goal. For fully acquiring the concepts of what was explained during tutorials on the cells of the immune system, the seminars were used to add an extra layer for students by finding out by themselves. For instance, groups of two students were made to sit together on one cell type of the innate immune system and to use ChatGPT to answer and present on what the immunological function of these cells were and how this was performed. For the various T-helper cells, a similar approach was followed, this time using Wikipedia. This approach was highly appreciated by the students. For the pathology part, where lectures for the whole cohort of students were used, interactive quizzes using Mentimeter (www.mentimeter.com) were used to assess whether students were well prepared for class. To gain insight into the various cell types in a disease context, dentistry-related cases were presented in an online microscopy practical. Each case was introduced with a YouTube clip, after which the students themselves could go through the microscopic slide online. Using slide annotations and more basic, molecular questions, the student could practice his/her knowledge during the cases of the microcopy practical with immediate feedback on the given answers.

### 2.5 Incorporate dentistry-related examples to support genetic learning on a weekly basis

One of the challenges of any course design in the biomedical field is to make it tailored for the student audience (11). For this course, one should find examples that are relevant to the field of dentistry. At the stage of course design, we selected six examples of dentistry topics that were included in the immunology part (Table 2), most of them case studies that could be brought down to a single gene defect. Table 2 shows that dentistry-related examples could be incorporated for each of the immune system aspects. A leading principle in immunology is that leukocytes have to find and migrate into the inflamed tissue. Leukocyte adhesion deficiency (LAD) is associated with periodontitis and students had to come up with arguments as to why this is associated with periodontitis as well as with skin ulcers in patients with LAD (12). An example of an inability to clear pathogens is Papillon–Lefèvre syndrome, where neutrophils without active cathepsin C are unable to make functional phagolysosomes, leading to juvenile periodontitis. Students were asked to come up with an explanation of the immune system defect after showing swollen gums and radiographs of a patient with Papillon–Lefèvre syndrome. It was also a good case to discuss the social consequences since patients wear complete dentures at an early age and can be stigmatized and unjustly associated with neglected oral healthcare (13). Candidiasis (introduced during Microbiology in Dentistry during year 1) is a phenomenon associated with mutated IL-17 receptors. This causes the Th17-helper cell population to lose functionality. The case study by Yakıcı et al. (14) helped repeat what was learned 1 week earlier on flow cytometry. To make immunology tangible, students should get an idea of how immunology is studied in a laboratory setting. A study from our own group on gingiva fibroblasts and Toll-Like Receptor (TLR) activation was used to show the parameters that are so logical for laboratory immunologists: classification of cells with flow cytometry, secretion of inflammatory cytokines IL-1β and TNF-α, and T-cell proliferation (15). IgA is probably the class of immunoglobulins that is of particular relevance to dentistry, since it is trafficked through the salivary gland epithelium into the saliva. Patients with IgA deficiency present with more caries, especially in the primary dentition (16). Students were asked to come up with explanations that were not provided in the article as to why the secondary dentition was not affected. Finally, dentists may experience more allergies to acrylate, the filling material that they are working with. It is also one of the components of nail extenders. A newspaper clipping of a professor at ACTA who is an expert on allergy (A. Feilzer) was the basis of a discussion as well as the basis of a more fundamental question: what type of allergy and how can it be envisaged that this allergy has antigen presentation as the basis, whereas acrylate is not a peptide. By incorporating these examples, the course should contribute to training dentists who are not following cookbooks or standard protocols to treat patients, but who have an awareness of certain immunological and pathological phenomena, as can be manifest in the dentist's chair. Thus, already early in their dentistry training, students gain knowledge and will contribute to a more personalized treatment plan.

**Table 2:** Examples of immunology and dentistry.

Dental case studies 1–6	Part of immune system/learning objective/reference
Case 1: Leukocyte adhesion deficiency	Example of *immune cell migration for all types of immune cells*
	Learning objective: general knowledge on the mechanism of diapedesis for immune cells to reach target tissue
	Dental problem: periodontitis
	Reference: Geroldinger-Simić et al. (12)
Case 2: Papillon–Lefèvre syndrome	Example of *innate immune system*
	Learning objective: link lack of cathepsin C deficiency to inability to clear pathogens in phagolysosome
	Dental problem: periodontitis
	Reference: Abdul et al. (13)
Case 3: Mutation in the receptor for IL-17	Example of *cellular immune response*
	Learning objective: how IL-17 receptor deficiency leads to candidiasis, recap of flow cytometry
	Dental problem: candidiasis
	Reference: Yakıcı et al. (14)
Case 4: Immunology in the lab	Example of *both innate and cellular immune system*
	Learning objective: familiarize with common laboratory approaches such as TLR activation, flow cytometric analysis of cell populations, T-cell proliferation, and cytokine measurements with ELISA
	Dental problem: mimic of chronic inflammation such as periodontitis
	Reference: Moonen et al. (15)
Case 5: IgA deficiency and caries	Example of *humoral immunity*
	Learning objective: deduce how IgA, as part of saliva, is important for control of biofilm, lack of it may lead to more caries
	Dental problem: caries
	Reference: Tar et al. (16)
Case 6: Allergy to acrylate nails	Example of *allergy*
	Learning objective: create clinical awareness how one dental material, acrylate, may progressively lead to allergic dentists, grossly affecting their career
	Reference: newspaper clipping.

### 2.6 Dentistry students should learn principles and should be lifelong learners and equipped with immunology and pathology knowledge to support their future practice

In addition to providing basic knowledge and incorporating examples of the immune system and dentistry, it is always important to ask the question: what part of the offered course is meaningful and additive to the anticipated career as a dentist? A deliberate choice was to invest, next to the textbook, in more explanation about the cluster of differentiation (CD) nomenclature. When reading academic dentistry literature, leukocyte composition and flow cytometric analysis are important topics. In addition, and this is an important link with pathology, it is important to introduce what tissue sections are and how one can determine immune cell composition with immunohistochemistry. Both techniques are based on CD presence and were introduced with a short introductory clip taken from YouTube, followed by a mind map. Students were placed in small groups and each group was asked to report on the meaning of one of the aspects of the mind map. ChatGPT was suggested as a way to find a quick answer, and slides were prepared by the teachers (TS and TdV) and were shown after this exercise. Such a flipped classroom approach was previously used to get students familiar with reading scientific literature (17).

### 2.7 Make it a logical educational journey

Finally, this was something we, as teachers, learned during the course: immunology and pathology, when explained in a logical sequence, are two topics that very well help in finding a kind of logic for the students. At the preparatory phase, the book *Immune* by Philipp Dettmer (18) was inspirational, since it succeeded in explaining the immune system to a lay audience without making use of technical terms and each chapter ended with a cliffhanger and logical follow-up. When adding specifics in a logical order, for instance when explaining the first major histocompatibility complex (MHC) and antigen presentation before the immunological synapse with T cells can be explained, it is important to regularly check whether the group is still aligned and still follows the intriguing lessons of immunology and pathology. It is also helpful to emphasize that a task of T-helper cells is indeed to *help* the existing innate immune cells. In the end of such a course, it will become immuno-*logical* and patho-*logical* for students.

## 3 Learning environment (setting, students, faculty), learning objectives, and pedagogical format

The course followed the didactic model that is generally applied to the new curriculum. Students meet in tutorials, seminars, and lectures, each for 2 h a week, resulting in 6 contact hours. In the final week of the course, team-based learning was applied to integrate all that was learned in an application session. Each week started with a tutorial where the cohort was split up into two groups of approximately 70 each. In principle, this would allow for more interaction than a lecture. The tutorials were on general principles of the innate immune system, cellular immune response, and humoral immune response. Tutorials were especially useful in weaving in the specific examples of dentistry (see Table 2) per aspect of the immune system.

Tutorials were followed up with seminars, explicating what was learned in tutorials. Within seminars of a maximum of 24 students per group, general principles were rehearsed, giving it another layer, by explaining newly learned concepts by yourself. As an example, the cells of the innate immune system were rehearsed in groups of approximately three to four students. Each group was given time to prepare on the cellular function of neutrophils, monocytes, NK cells, etc. Sources could be the book, but ChatGPT as well. Students and teachers found that ChatGPT was extremely useful also in summarizing general overarching principles, which are sometimes spread over more than one chapter in the textbook. Examples are: “Explain the function of the immune synapse” and “What is meant by co-stimulation in the context of immunology?” To incorporate other and more proven sources, the same exercise was repeated in the second week for the T-helper cells but then using Wikipedia. Seminars were also extremely useful in trying to further categorize cell functions. As an example, for the innate immune system there are essentially two ways of elimination: phagocytosis or lysis of the bug by making holes (perforin) and degradation (granzyme). For the helper cell exercise, an overarching message was that they are indeed there to help.

As a third teaching method, a lecture on the tissue reaction (pathology) was taught at the end of the week, where the basic principles and the molecular aspects of acute inflammation, chronic inflammation, and tissue repair were discussed. During the lecture on chronic inflammation, in particular, the transition from innate to specific immune response was clearly explained.

The fourth teaching method was team-based learning, where one theme, the autoimmune disease Sjögren’s syndrome, was used as an integrative topic for both the immunology and pathology lectures. Team-based learning should encourage students to practice academic reasoning. In addition, it provides an ideal opportunity to face students with course-overarching aspects. We chose an application session that contained choices of immunohistochemical stainings of the inflammation area to elucidate the cellular and functional composition of cells, a question that connected Sjögren’s syndrome to the prevalence of periodontitis, and a question on biologicals such as anti-TNF-α and anti-B-cell medication (7).

Finally, during the 4-week course, to acknowledge the self-set limitations of the course, students could choose a topic of choice for a more in-depth search. By formulating a research question or aim on the given subject, and then searching, selecting, and reading literature, they deepened their understanding of a particular subject. Structuring their essay, a section of their essay and synthesizing conclusions trained academic skills while stimulating the integration of basic principles taught during the course with details found in scientific literature.

## 4 Results to date/assessment (processes and tools: data planned or already gathered)

For every newly developed course, one should monitor how the course comes across to students early during the course. When doing so, one can adapt to the suggestions put forward by the students. The year representative was asked near midway to assess this, which was simply done by sharing a Google Docs form with all participating students. Questions were about teachers (which were OK), but also on the level of the course (it was perceived as difficult, with a lot of new and hitherto unknown topics) and on whether immunology and pathology were logical partners (which was the case). Based on the feedback that the course was perceived as difficult, term glossaries were made per week in the “What do we have to know per week” documents. These were put on the digital learning platform. These glossaries helped define to what extent the immunology part should be learned.

The exam represented an equal distribution of questions per week, two-thirds for Immunology and one-third for Pathology. Two-thirds of the questions were multiple-choice questions (mainly on knowledge), and one-third were open questions that were of the higher Bloom levels (understanding, interpreting).

Both the course (*n* = 77) and the exam (*n* = 40) received a relatively high percentage of student feedback, probably due to the message we, as teachers of the course, put to the students: “We very much value your feedback, especially since it is a new course. You are the pioneers. You have a responsibility to respond, since together (students and teachers) we can make education at ACTA better.” This high response rate was achieved by sending three messages on the digital learning environment and by providing an extra opportunity during the next course to fill out the evaluation in class.

Overall, the course scored high on the Likert scale of 1–5 points (>4 for most categories, with a general appreciation of the course of 4.4) (Table 3). We acknowledge that the interpretation of the table is purely descriptive, but the grading of educational and general aspects for such a new course is essential for assessing whether the course was successful. When implementing a new curriculum with an emphasis on novel, activating teaching and learning methods, it is important to monitor whether they are meaningful. Questions on the educational methods (lecture, seminars, practical, group engagement, team-based learning, the role of the teacher as coach) all received high scores. This means that, apparently, this course fits naturally into how ACTA wants the various educational methods to be used. They made sense to the students in their learning process. Specific responses by the students on the appropriateness of activating learning and teaching were as follows: “The various methods of education were nice” and “I especially appreciated the seminars. The interactions with the teacher and with the fellow students enabled me to understand the material.” When considering the educational journey, it was mentioned: “It is very interesting to have lessons in a logical sequence. It was a very interesting course and very good at interactive teaching. Seminars were useful.”

**Table 3:** Student course evaluation, with emphasis on the appropriate use of the various educational methods and general course aspects.

Questions	1–5 Likert (1 = strongly disagree, 5 = strongly agree)					Average *N* = 77
	1	2	3	4	5	
Educational methods						
The teacher succeeded in positioning him/herself as coach.	0	2	11	30	34	4.25
The lectures inspired me.	0	0	9	39	29	4.26
The activating methods used in the seminars promoted interaction with fellow students and the teacher, allowing me to apply the material better.	0	1	5	31	40	4.43
The seminars have encouraged me to actively work on the study material.	0	1	8	23	45	4.45
The pathology digital microscopy practical was useful (*n* = 76).	2	4	17	31	22	3.88
Working in a group has positively contributed to my learning process.	1	5	25	25	21	3.78
TBL is a useful addition to other educational methods.	1	4	8	29	35	4.21
Working according to the TBL method has given me in-depth insight into the course material.	2	4	10	36	25	4.01
General aspects of the course						
The course material (texts, slides, assignments, clips) were clear and informative.	0	3	5	34	35	4.31
The course was well organized	0	1	8	23	45	4.45
I have learned a lot during this course.	0	0	1	31	45	4.57
During this course I have worked on my academic development.	1	1	24	34	17	3.84
I learned a lot in this course because of the effort of the teacher.	1	1	8	25	42	4.38
The exam was a good indicator of what I have learned (*n* = 40).	0	2	1	21	16	4.28
The teaching and the exam were a good fit (*n* = 40).	0	1	3	16	20	4.38
I felt free and safe to ask questions and express my opinion during teaching.	1	1	8	18	49	4.47
In summary, my overall appreciation for this course was (1–5).	0	0	6	34	37	4.40

Similarly, the general aspects of the course (course materials, course organization, learnings, exam reflected what was learned during the course, save learning environment) all scored high.

Regarding team-based learning, students responded to the open question on what they learned during TBL: “The relationship between immunology and pathology became clear after TBL.” Also: “The application session about Sjögren leads to novel insights. Application of knowledge at the level that is relevant for dentistry. I learned a lot about autoimmunity.” About the group work during TBL, it was said: “I liked it that everyone participated in one session, very good discussions were taking place and everyone was motivated,” and “It was nice to do the application session in one cohort.”

## 5 Discussion on the practical implications, objectives, and lessons learned

Overall, in modern dentistry and medical curricula, there is a clear tendency to reduce the number of lectures in favor of more activating teaching and learning methods, for instance in smaller groups (19). Teaching smaller well-prepared (by self-study) groups in seminars led to more satisfaction as reported by students but did not automatically lead to better learning results (20). When comparing formal lectures to less formal but interactive ones, the interactive ones were more popular and led to better grades (21).

Here, we show the design of a course that contained all elements in the appropriate weight as laid down in the framework of the new Bachelor curriculum at ACTA. It contained tutorials, seminars, lectures, a practical, and a team-based learning session, largely following the didactic model used to implement the new curriculum. Separately, all items were appreciated by the students and well-rated. It was also mentioned that the diversity of educational methods contributed to the learning of the students.

For clarity reasons, we decided to allocate tutorials and seminars to the immunology contact hours, the lecture hall lectures, and the practical to the pathology contact hours. For the immunology part, it was a pleasant surprise to us, teachers, to experience that immunology, which requires a specific build-up, benefits from such activating and interactive educational formats. Likewise, the pathology lectures and the digital microscopy were seen as complementary to each other.

To try to explain immunology as an educational journey was very much inspired by Philipp Dettmer's awe-inspiring book *Immune*. He takes the reader by the hand in a very clear stepwise approach, avoiding technical terms and using good, helpful metaphors, such as: if an immune cell is the size of a man, then a bacterium is the size of rabbit, and macrophages—they eat a lot—are the size of rhinoceroses. These metaphors are extremely helpful when explaining how the body then copes with parasites that are too large to be phagocytosed. What solutions are available? Make holes! Immunology, when told as an ongoing story with cliff hangers and with well-chosen examples tailored to the dentistry field, has the potential to fascinate an audience of dentistry students. It connects to pathological processes at the tissue level, such as wound healing and acute and chronic inflammatory processes.

## Data Availability

The original contributions presented in the study are included in the article/Supplementary Material, further inquiries can be directed to the corresponding author.
